# Depressive Symptom Networks in Rheumatoid Diseases

**DOI:** 10.1002/hsr2.71164

**Published:** 2025-09-17

**Authors:** Elizabet Ukolova, Marie Hanková, Michala Lustigová

**Affiliations:** ^1^ Faculty of Science, Department of Demography and Geodemography Charles University Prague Czechia; ^2^ Interdisciplinary Centre on Population Dynamics (CPop) University of Southern Denmark Odense Denmark; ^3^ Faculty of Science, Department of Social Geography and Regional Development Charles University Prague Czechia

**Keywords:** comorbidity, depressive symptoms, network, rheumatoid diseases, SHARE

## Abstract

**Background and Aims:**

Patients with Rheumatoid diseases (AR) are at higher risk for depressive symptoms. This study aims to identify key depressive symptoms that lead to the accumulation of additional symptoms over time, using Survey of Health, Ageing and Retirement in Europe (SHARE) data from year during the follow up period 2015–2019/2020 (sample size 32,082 individuals).

**Methods:**

We applied network analysis, where nodes represent depressive symptoms by EURO‐D scale and edges are weighted by odds ratios. Changes in symptom relationships were analysed by transitions between health states: “healthy,” “AR only,” and “AR with additional comorbidity.”

**Results:**

To prevent further depressive symptom accumulation in AR patients, addressing irritability and lack of interest is crucial. In males, loss of appetite should also be targeted, while in females, depressive feelings need specific attention. Males with AR and additional comorbidities show a significant increase in symptom network density, while females have stronger associations with interest, appetite, guilt, and depression.

**Conclusion:**

AR is associated with significant changes in the structure of depressive symptom networks, varying with health progression. Attention to irritability, lack of interest, appetite, and depressive feelings is crucial for preventing further mental health declines in newly diagnosed AR patients.

## 1 Introduction

Rheumatoid diseases, commonly referred to as arthritis (AR), encompass a broad group of chronic, progressive autoimmune disorders that affect the joints and muscles, leading to significant mortality and morbidity [1, 2]. These conditions also severely impact quality of life due to the persistent and intense pain experienced by AR patients [3, 4]. Approximately 20% of adults in the USA have been diagnosed by a doctor with some form of AR. The most common form, rheumatoid arthritis, affects approximately 0.38%–0.78% of Europeans [5, 6, 7], increasing to around 2% in the elderly population [8]. Additionally, more than half of individuals diagnosed with AR experience mild to severe depressive symptoms [9, 10], nearly twice the prevalence observed in the general population [11, 12].

The strong connection between AR and mental health presents both opportunities and challenges for disease progression, as the mere perception of the severity of the disease plays a role in its outcome [13]. The AR patients with generally pessimistic attitudes and more severe depressive symptoms are likely to experience faster disease progression [14, 15]. Furthermore, as demonstrated by Englbrecht et al. [16], AR patients who have greater concerns about their future—such as decreased independence or financial issues due to work absenteeism related to AR—tend to experience more pronounced physical symptoms of the disease [14]. Other studies explain the connection between AR and mental health through a biological basis. In simple terms, AR causes cytokine reactions, which have the potential to activate neurochemical responses, ultimately manifesting as depressive symptoms [10, 17].

The impact of AR on an individual's quality of life is extensive: increased pain, fatigue, limitations in daily activities or mobility, sleep problems and dealing with medication side effects [4, 18, 19, 20]. All of this can impact mental health in very different ways; however, most existing studies primarily focus on the quantitative differences in depressive symptoms between individuals with AR and the general population. The dimensions of self‐reported mental health that change with the onset of AR remain largely unexplored. However, a qualitative understanding of AR's psychological manifestations could help improve the effectiveness of mental health support for patients with AR. Furthermore, current studies that focus on mental health of AR patients are often cross‐sectional, making it impossible to track how mental health evolves over the course of living with AR [21]. This paper contributes to filling these knowledge gaps by describing changes in the architecture of relationships between individual depressive symptoms, classified according to the EURO‐D scale, over a 5‐year period. The aim is to identify the key symptoms that significantly increase the risk of developing additional depressive symptoms, ultimately leading to higher overall depressive scores. The analysis is done within subpopulations by health status, focusing on transitions between health states “healthy (not diagnosed AR),” “with AR only” and "with AR and additional comorbidity.”

Since we work with data from the *Survey of Health, Ageing and Retirement in Europe* (SHARE), we rely on self‐reported diagnosis of AR, as well as self‐reported comorbidities. During the SHARE interview, respondents are asked to report whether a doctor has ever told them that they have any of following conditions: Rheumatoid Arthritis, Osteoarthritis or other rheumatism. The list of diagnoses included as comorbidities is provided in the Appendix.

## 2 Materials

The SHARE is a multidisciplinary, panel‐based study, which collects data about physical and mental health, socioeconomic status and social and family networks among people aged 50 years and more. The data is collected in waves, starting with the first Wave in 2004, followed by eight additional waves at 2 to 4‐year intervals [11, 22, 23]. The survey is designed as refreshed panel study, meaning that 20% of the sample is regularly supplemented, especially with respondents aged 50. An eligible respondent is an individual over the age of fifty and their partner (regardless of age) [24].

In this paper, we use data from waves 6 and 8, which included 28 European countries: Austria, Belgium, Bulgaria, Croatia, Cyprus, Czechia, Denmark, Estonia, Finland, France, Germany, Greece, Hungary, Ireland, Italy, Latvia, Lithuania, Luxembourg, Malta, the Netherlands, Poland, Portugal, Romania, Slovakia, Slovenia, Spain, Sweden and Switzerland and Israel. The interviews for the wave 6 were conducted during February to November 2015 and for the wave 8 from April 2019 to March 2020 [25]. Our sample includes respondents who participated in both waves, totaling 32,082 individuals. Among them, 30,084 responded to both mental health interview and physical health interview in both waves. Consequently, the drop out was around 6%. The final sample we work with consists of 42% of males and 58% of females.

Depressive symptoms are measured using the EURO‐D scale, a widely used and validated instrument for studying elderly populations, as well as for making international comparisons [26, 27, 28, 29, 30]. The EURO‐D scale consists of 12 items: *depressed mood, pessimism, suicidality, guilt, sleep, interest, irritability, appetite, fatigue, concentration, enjoyment* and *tearfulness*. The EURO‐D questionnaire is provided in the Appendix.

Physical health is measured by asking respondents if a doctor has ever told them that they have been diagnosed with any of a specified set of diseases, including rheumatoid arthritis, osteoarthritis, or other forms of rheumatism. Clearly, the data on disease presence or absence is self‐reported.

As mentioned earlier, our goal is to compare the changes in the structure of interrelations between depressive symptoms across groups categorized by transitions between health states during 2015–2019/2020, with a focus on AR. For that purpose, the following health states are distinguished: (i) healthy (no reported chronic condition), (ii) has only AR, (iii) has only single other disease (no AR), (iv) has AR and any additional comorbidity, (v) has multiple diseases, excluding AR. The frequencies of all transitions between defined health states are shown in Table 1. The most frequent transition involving AR at any stage was remaining in the state with AR and comorbidity in both the 2015 and 2019/2020 waves. In this transition, females dominated, accounting for 75%.

**Table 1:** Frequencies of transitions between health states during 2015–2019/2020, in brackets: percentage of females, rows: health state in 2015, columns: health state in 2019/2020.

	No disease	AR	Other disease	AR and comorbidity	Other disease and comorbidity	Total
No disease	3758 (56.4%)	306 (68.3%)	1894 (53.6%)	476 (64.7%)	955 (46.6%)	7389
AR	186 (64.5%)	268 (68.7%)	174 (66.1%)	323 (73.4%)	74 (70.3%)	1025
Other disease	1222 (55.6%)	187 (64.2%)	3008 (53.5%)	1109 (63.0%)	2207 (49.3%)	7733
AR and comorbidity	213 (64.8%)	164 (76.8%)	695 (65.8%)	3340 (75.0%)	1439 (66.5%)	5851
Other disease and comorbidity	377 (56.5%)	59 (71.2%)	1337 (52.0%)	1597 (63.4%)	4716 (48.4%)	8086
Total	5756	984	7108	6845	9,391	30,084

Abbreviation: AR, arthritis.

## 3 Methods

First, for each respondent, we identified the transition between health states they experienced during the follow‐up period. Next, within each group defined by the transition in health state, we used odds ratios to measure the strength of associations between depressive symptoms separately for wave 6 and wave 8. The odds ratio (OR) is calculated using the following formula [31, 32]:

$$\mathrm{OR}=\frac{\frac{N_{i,j}}{N_{\neg i,j}}}{\frac{N_{i,\neg j}}{N_{\neg i,\neg j}}},$$

Where *N* represents the number of individuals in each group based on transitions in health states, and indices *i* and *j* indicate the presence or absence of depressive symptoms. For example, *N*_*i,j*_ equals to the number of individuals having both symptoms *i* and *j*. Similarly, *N*_*¬i,j*_ stands for the number of individuals without symptom *i* but with symptom *j*. To calculate the confidence intervals for the OR, we start with calculation of the standard error using following expression [32, 33]:

$$\sigma =\sqrt{\frac{1}{N_{i,j}}+\frac{1}{N_{i\neg ,j}}+\frac{1}{N_{\neg i,j}}+\frac{1}{N_{\neg i,\neg j}}},$$

Assuming normal distribution of the standard error, we can calculate the confidence intervals of OR using the formulae:

$$\mathrm{CI}\left(\mathrm{OR}\right)=\text{exp}(\text{ln OR})\pm z_{\alpha /2}\times \sigma ,$$

Where *z*_*α*/2_ is the quantile of the standardized normal distribution corresponding to the confidence level, which in this case was considered to be 95%. An OR greater than 1 indicates that the odds of the event occurring are higher in the exposed group compared to the nonexposed group. Here, it means that the chance of experiencing depressive symptom *j* is higher in the group that reports having symptom *i*, compared to the group that does not report having that symptom. In the Appendix, we provide all the OR along with their corresponding confidence intervals, calculated for both wave 6 and wave 8.

When the strengths of relationships between all possible pairs of symptoms were quantified, networks of depressive symptoms were created. These networks are composed of nodes (representing depressive symptoms) connected by edges. Depending on the application, edges can connect all nodes to create a full network or only connect nodes if prespecified conditions are met. Here, nodes are connected, if more than 20 respondents reported having the pair of the depressive symptoms. Additionally, the importance of edges in the networks can be determined by assigning them a weight, creating weighted networks. Here, we use two different edge weights. Firstly, the weights represented the ratio of the OR at the wave 8 to that at the wave 6. This means that higher weights are assigned to edges connecting depressive symptoms that were more strongly associated at the end of the follow‐up compared to the beginning. Secondly, the weights corresponded to the OR. If the OR was not statistically significantly different from unity, indicating no association between depressive symptoms, the edge weight was set to zero.

Networks not only offer an effective way of visualizing relationships between objects, but they also allow for the analysis of roles of nodes from various perspectives. The benefit of network analysis lies in its ability to describe the relationships between nodes within the complex architecture of connections, rather than focusing solely on pairs of symptoms in isolation from the others.

To describe the roles of nodes, we use node strength, which is given by the sum of the weights of edges that are being incident on the node. In this case, we use the second type of weights, namely OR significantly higher than 1. The node strength is calculated separately for wave 6 and 8.

The statistical analysis was conducted using SAS software version 6.4, and the visualizations of the networks were created in R Studio version 4.3.1.

## 4 Results

The overall prevalence of depressive symptoms differed notably between the AR and non‐AR populations. In the non‐AR group, nearly 25% reported no depressive symptoms (EURO‐D = 0) in both Waves 6 and 8, compared to only 12% in the AR group. In contrast, around 12% of the non‐AR population reported five or more depressive symptoms, while nearly one in four individuals with AR did so (EURO‐D > 4).

**Figure 1:**
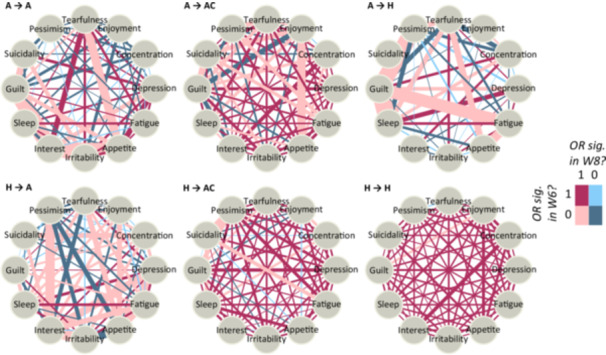
Networks of depressive symptoms in groups with arthritis in wave 6 (first row) and in healthy group in wave 6 (second row), females.

**Figure 2:**
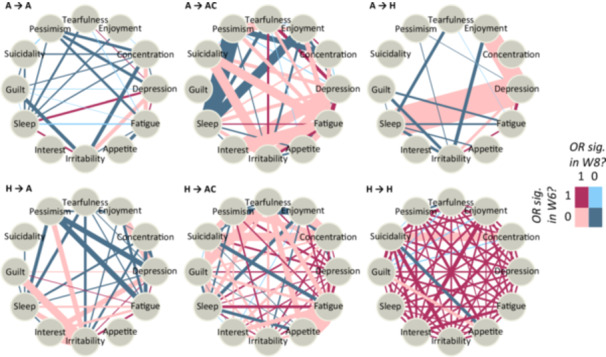
Networks of depressive symptoms in groups with arthritis in wave 6 (first row) and in healthy group in wave 6 (second row), males. A = has only arthritis; AC = has arthritis and additional comorbidity; H = healthy (no disease).

Figures 1 and 2 show the networks of depressive symptoms by sex for distinct groups, categorized by the change in health status they experienced between wave 6 and wave 8. Transitions from the “AR and comorbidity” health state are detailed in the Appendix. The colors of the edges represent the significance of the associations between the symptoms in both waves. The widths of the edges indicate the ratio of the ORs between wave 6 and wave 8. The bolder the edge, the greater the increase in the association between the depressive symptoms. Not all edges are present in some of the networks because the co‐occurrence of the symptoms it connects was negligible (< 20). Additionally, while Figure 1 depicts *depression* as one of the symptoms, it represents a *depressive mood* rather than a formal diagnosis of depression.

Focusing firstly on the groups which reported same health state in both waves, we see especially in females large level of stability in the group having AR and comorbidity in both waves (ACAC) (see Appendix), as well as in the group reporting having no medical conditions in both waves (HH). Depressive symptoms are predominantly significantly associated in both waves. In males, in the group HH there was an increase in OR between *concentration* and *suicidality*, resulting in significant association between these symptoms.

Getting the diagnosis of AR is accompanied by considerably less stability in the relationships between depressive symptoms. In the female group that acquired arthritis but reported having no medical conditions in the first wave (HA), the ORs with *depression, concentration, enjoyment, interest, appetite*, and *pessimism* significantly increased. The results are similar for males, except for the specific depressive symptoms experiencing the change. In males, *irritability* and *guilt* were the dominant symptoms involved in the change. Interestingly, in females, the association between *irritability* and *guilt* remained similar in both waves in the HA group.

For the groups that already had AR in the first period and reported it either alone (AA) or with another comorbidity in the second period (ACA), the interconnectedness between depressive symptoms also increases. In the AA group, there is a notable further increase in the association with the symptom *interest*. In contrast, the changes in the strength of relationships in the ACA group are not concentrated around a single symptom. However, females in the ACA group tend to have weaker associations and lose the significance of edges related to symptoms such as *irritability, appetite*, or *enjoyment*. In males, the corresponding networks are sparser, and the associations between symptoms are mostly nonsignificant in both waves. However, in the AA group of males, there are increases in ORs for *depression* and *irritability*, as well as for *concentration* and *fatigue*. In the ACA group of males, rising ORs are observed for *irritability* and *fatigue*.

Interestingly, in males, the development of comorbidities in addition to AR is associated with significant changes in the architecture of connections between depressive symptoms. For instance, pairs involving *fatigue, depression, irritability*, or *suicidality* experience an increase in OR and become significantly associated by wave 8.

Finally, there are two groups who “heal” from either arthritis alone (AH) or from arthritis and some other medical condition (ACH). This “healing” is accompanied by a rapid increase in associations involving *suicidality, sleep, irritability, pessimism, fatigue*, and *appetite* in females. In males, the strengthening of associations is observed in pairs with *depression* and *sleep* in the AH group, and with *depression, irritability*, and *guilt* in the ACH group. We can hypothesize that “healing” from AR or from AR and its physical comorbidities might signal nonresponse, which could also be indicative of worsening mental health.

**Figure 3:**
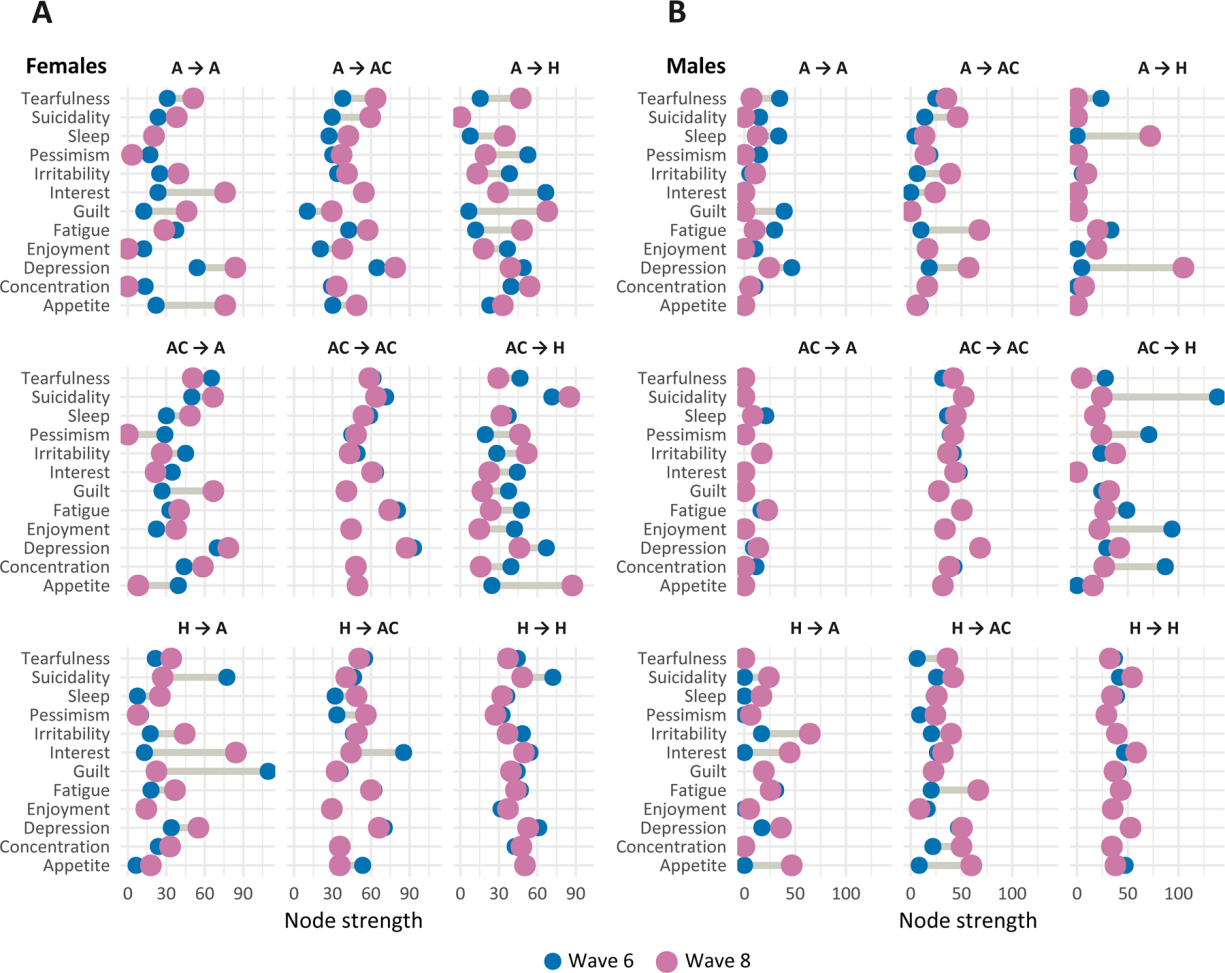
Changes in node strength in networks of depressive symptoms, by sex and transition between health states. A = has only arthritis; AC = has arthritis and additional comorbidity; H = healthy (no disease).

Figure 3 shows the changes in node strength between wave 6 and wave 8, separately for males and females and subpopulation by transition between health status. The node strength is equal to the sum of significant OR. The extent of the change in the node strength is given by the distance between two points, the pink one (i.e., for the wave 8) and the blue one (i.e., for the wave 6).

Receiving a diagnosis of AR is closely associated with a substantial increase in the node strength of *interest* and *irritability*. In males, a similar increase is observed in *appetite*, while in females, it is seen in *depression*. However, the change in node strength for depression between waves 6 and 8 is considerably less pronounced compared to the previously mentioned symptoms. Overall, *irritability* and *interest* stand out as key symptoms for the development of additional depressive symptoms when a person transitions from being healthy to being diagnosed with AR. Specifically for females, the transition in the HA group is also marked by a significant decrease in the strength of associations with *guilt* and *suicidality*.

There are also several noticeable changes in the ACA and AA groups. In females, *interest, appetite, guilt*, and *depression* gain importance, which is not observed in males. For them, the ACA group shows an increase in the strength of *fatigue* and *appetite* symptoms, while this is not the case for females.

Interestingly, substantial changes in node strength are observed in the group of respondents who report being healthy in the follow up wave, despite not being healthy in the staring one. Precisely, in females in the ACH group, *appetite, pessimism*, and *irritability* become the most central nodes. In contrast, males in this group predominantly show decreases in the strength of *suicidality, enjoyment*, and *concentration*. In the AH group, females exhibit the most pronounced increases in *guilt* and *fatigue*, whereas in males, the changes are driven by a rapid increase in the strength of *depression* and *sleep*. This indicates, that shifts in node strength in ACH and AC groups are highly sex specific.

## 5 Discussion and Conclusion

In this paper, we aimed to explore the evolution of the structure of relationships between depressive symptoms in populations experiencing transitions between three health states: (i) being healthy, (ii) having AR, and (iii) having AR with additional comorbidity. First, we analyzed changes in the odds ratios (OR) between nodes. Next, we identified the nodes that gained the most strength as individuals progressed through predefined health states.

Our findings can be summarized into several points:

1. Staying with both the most conditions (ACAC) and healthy (HH) is associated with stability in the structure of relationships between depressive symptoms.
2. Developing AR after being healthy (HA) is linked to increases in the odds ratios between depressive symptoms. Specifically, *irritability, interest*, and *appetite* in males, and *depression* in females, become the most central nodes, thereby increasing the risk of accumulating further depressive symptoms in patients with AR.
3. In males, the networks substantially increase in density when additional comorbidity is added to AR (group AAC), which is not observed in females. In females in the AA and ACA groups, there are increases in the strength of associations for *interest, appetite, guilt*, and *depression*.
4. Reporting no medical conditions in the follow up wave after having AR or AR with comorbidity is associated with strengthened associations between depressive symptoms. The changes in node strength are gender‐specific and can be summarized as follows: in males in the AH group, there is an increase in *sleep* and *depression*; in females in the AH group, there is an increase in *guilt, fatigue*, and *tearfulness*. In males in the ACH group, there is a rapid decrease in associations with *suicidality, enjoyment*, and *concentration*.

Although our paper is not designed to measure causality, we still observe that changes in health status, specifically receiving a diagnosis of AR, are accompanied by remarkable changes in the structure of dependencies between depressive symptoms. However, if individuals had AR and additional comorbidities in both waves, these changes are less pronounced, and the strength profiles of the nodes are very similar to those in the group reporting no conditions in both waves. On one hand, this suggests the importance of one's perception of AR and its impact on one's life. The main stress factor strengthening the relationships between depressive symptoms could be receiving the diagnosis. The ACAC group was not exposed to this factor during the observational period. On the other hand, the result could also indicate a lower willingness to respond among the ACAC group, possibly due to overall poorer health caused by both presence of additional diagnoses and living longer with AR for a longer period of time compared to HA or HAC groups. Additionally, the ACAC group might be more likely to drop out, which all could lead to lower accuracy of estimates of associations in the ACAC group.

Another contribution of our study is that we identified depressive symptoms that bridge the connections within the network. This finding is particularly important when designing preventive strategies against the development of severe depressive symptomatology, as it highlights which symptoms increase the risk of accumulating additional ones.

We identified some gender‐specific changes, particularly in the group with single AR in the first wave but who developed an additional comorbidity by the second wave. These findings might have several explanations. Firstly, males and females might experience different progression of AR. Secondly, males and females might react differently to the progression of AR. The former is widely discussed in the literature: females are known to have higher disease activity, lower response to therapies, and more frequent comorbid conditions [34, 35]. However, it is the male group that experiences major changes in the strength of depressive symptoms, and this is not because females already had high strength in wave 6. This suggests that in females, depressive symptoms are more associated with AR, whereas in males, AR contributes to increased interconnectedness between depressive symptoms.

Lastly, we showed that not reporting AR after having it in the first wave is ambivalent. On one hand, the strength of connections within the networks increases, for instance, in AH females. On the other hand, it shows remarkable decreases, namely in ACH males. We argue that the decreases specific to males do not symbolize improvement in either physical or mental health, but rather rising apathy in completing the questionnaires, which itself might be a signal of depression in the elderly (Koyama et al., 2014). In this light, males with AR and additional comorbidity are seen as the most vulnerable population.

There are several limitations to our study. Firstly, we did not include any variables that could impact the differences between groups by health status transition or to the differences in sex, such as age, age at diagnosis, socioeconomic variables, medication or country of residence. Especially the treatment a patient with AR is undergoing (including medication) could strongly affect the manifestation of depressive symptoms, and in fact, change in medical treatments during the follow up may be an important source of differences between groups by health state transitions. This could explain a decrease in the strength of associations between some of the symptoms during a “deterioration” in health status, typically, for example, in individuals who were previously healthy, but had AR at the end of the follow‐up, or in the ACA group. Thus, the reduction in the strength of associations between depressive symptoms could be a side effect of medical treatment AR.

Secondly, in wave 8, the data collection period ended in March 2020, shortly after the outbreak of the COVID‐19 pandemic. Although data collection was halted due to the pandemic, 6% of respondents still provided their data during March 2020, which may have introduced some biases into the interviews. Thirdly, we must stress that the health states were classified based on self‐reported medical conditions, which may not align with opinion of the medical professional. The health indicators used in this paper only focus on a limited range of diagnoses included in the SHARE survey. As a result, certain comorbidities, both physical and mental, may be overlooked, potentially leading to misclassifications of respondents' health status. For example, individuals in the “only AR” group could have additional diseases that were not surveyed during the SHARE interview, which could introduce bias. On the other hand, SHARE does include the most common chronic diseases in Europe. One of the other simplifications in this study is that the stage of AR and its severity are not monitored.

To conclude, AR could be associated with significant perturbations in the structure of dependencies between depressive symptoms. To mitigate further accumulation of depressive symptoms in individuals diagnosed with AR, it is important to focus on increasing awareness and intervention for irritability and lack of interest. In males, attention should also be given to loss of appetite, while in females, depressive feelings should be specifically addressed.

## Author Contributions

**Elizabet Ukolova:** conceptualization, investigation, writing – original draft, methodology, validation, visualization, writing – review and editing, software, formal analysis, project administration, data curation. **Marie Hanková:** writing – original draft, writing – review and editing, data curation. **Michala Lustigová:** funding acquisition, supervision, writing – review and editing, resources.

## Ethics Statement

This study is based on secondary analysis of anonymised data from the Survey of Health, Ageing and Retirement in Europe (SHARE). The SHARE data collection procedures have received ethics approval from the relevant research ethics committees or institutional review boards in all participating countries and are subject to continuous ethics review. SHARE‐ERIC's activities related to human subjects research are guided by international research ethics principles, including the Respect Code of Practice for Socio‐Economic Research and the Declaration of Helsinki (last revised at the 64th WMA Meeting in Fortaleza, Brazil, in 2013). The data are fully anonymised and comply with the General Data Protection Regulation (GDPR) of the European Union. Access to the data is granted only to registered researchers for scientific purposes under strict conditions defined by the SHARE data access agreement. All SHARE users are expected to be familiar with fundamental research ethics principles and to apply them appropriately in their work. Therefore, no additional ethical approval was needed for this secondary data analysis. Further information on SHARE's ethical and data protection framework is available at: https://share-eric.eu/data/data-access/conditions-of-use Ethical approval and consent were handled by SHARE (Survey of Health, Aging, and retirement in Europe) and details can be found at http://www.share-project.org. The SHARE study was reviewed and approved by the Ethics Council of the Max Planck Society. In addition, the country implementations of SHARE were reviewed and approved by the respective ethics committees or institutional review boards whenever this was required. The study was performed in accordance with relevant guidelines and regulations.

## Conflicts of Interest

The authors declare no conflicts of interest.

## Transparency Statement

The lead author Elizabet Ukolova affirms that this manuscript is an honest, accurate, and transparent account of the study being reported; that no important aspects of the study have been omitted; and that any discrepancies from the study as planned (and, if relevant, registered) have been explained.

## Supporting information

Revision 2 Appendix.

## Data Availability

The data used in the analysis are available at the SHARE website (https://share-eric.eu/data/). Authors calculations are available in the online repository: https://github.com/ukolovae/RA_and_depressive_symptoms.
