# Supplementary material for: Depressive Symptom Networks in Rheumatoid Diseases
Source: Health Sci Rep. 2025 Sep 17;8(9):e71164. doi: 10.1002/hsr2.71164 (PMC12441587; doi:10.1002/hsr2.71164)
Supplement: Supplementary file 1 — Revision 2 Appendix. [file HSR2-8-e71164-s001.docx]

# **Appendix**

**1. List of comorbidities**

In the SHARE survey, respondents were asked to report any of the following morbidities:

1. A heart attack including myocardial infarction or coronary thrombosis or any other heart problem including congestive heart failure
2. High blood pressure or hypertension
3. High blood cholesterol
4. A stroke or cerebral vascular disease
5. Diabetes or high blood sugar
6. Chronic lung disease such as chronic bronchitis or emphysema
7. Cancer or malignant tumour, including leukaemia or lymphoma, but excluding minor skin cancers
8. Stomach or duodenal ulcer, peptic ulcer
9. Parkinson's disease
10. Cataracts
11. Hip fracture
12. Other fractures
13. Alzheimer's disease, dementia, organic brain syndrome, senility or any other serious memory impairment
14. Other affective or emotional disorders, including anxiety, nervous or psychiatric problems
15. Rheumatoid Arthritis
16. Osteoarthritis, or other rheumatism
17. Chronic kidney disease
18. None
19. Other conditions, not yet mentioned

**
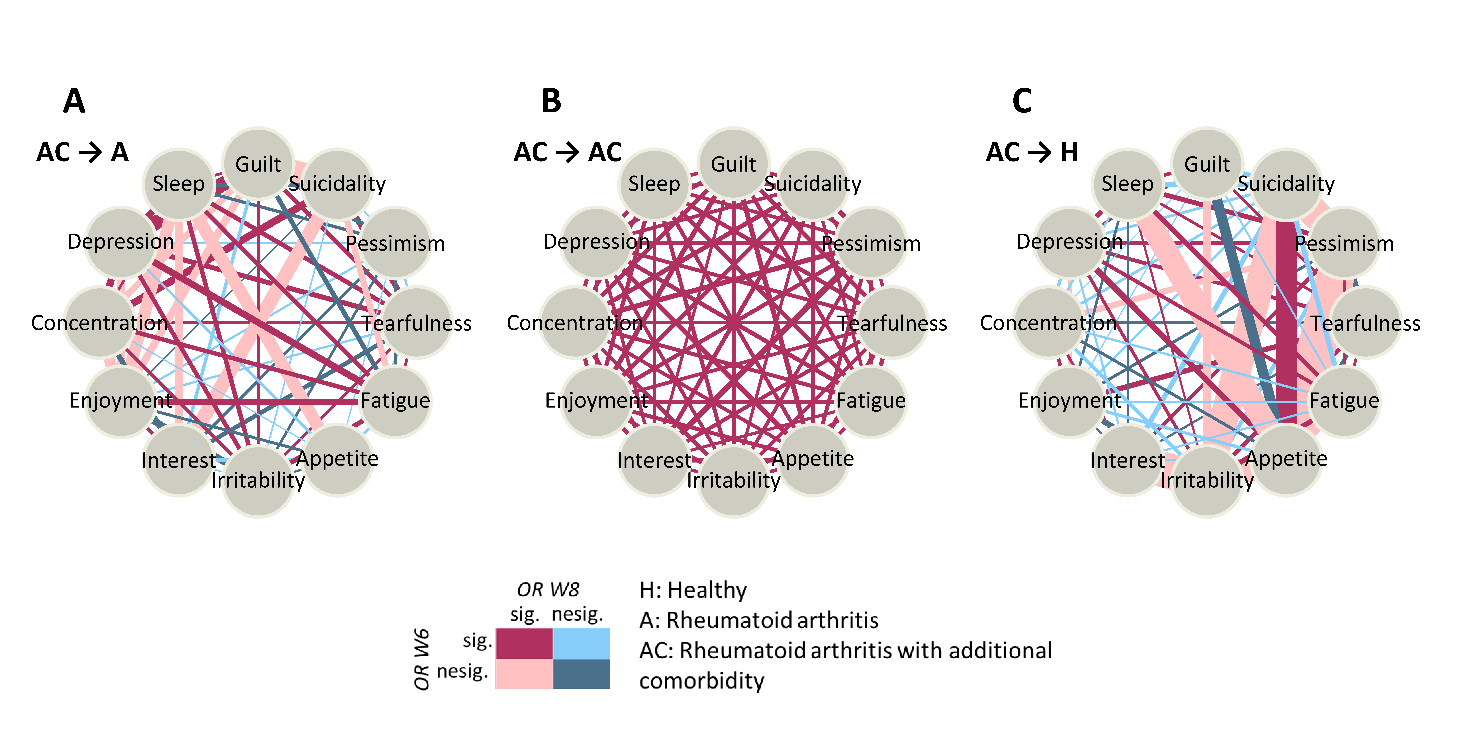
2. Figure: Networks of depressive symptoms in groups with Arthritis and comorbidity in wave 6, females**

**
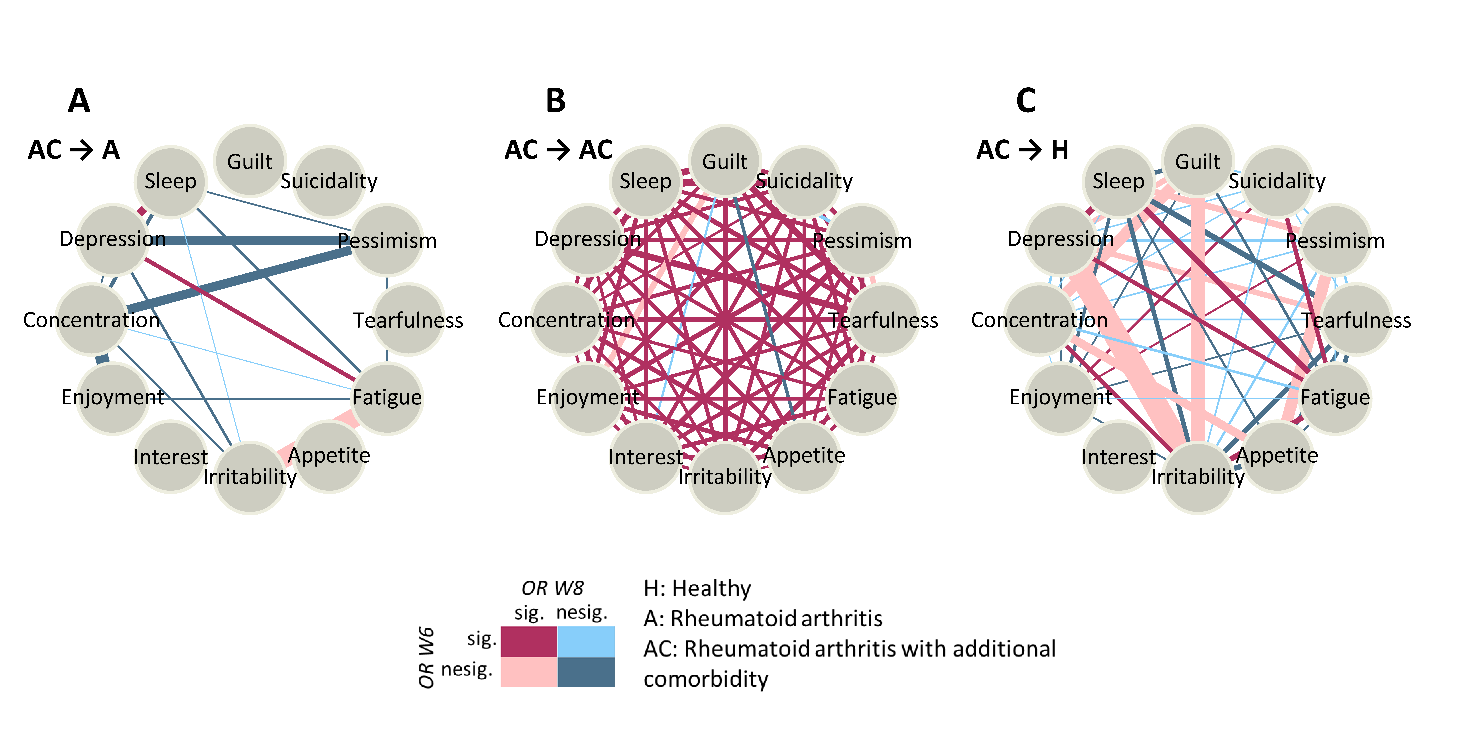
3. Figure: Networks of depressive symptoms in groups with Arthritis and comorbidity in Wave 6, males**

**4. The EURO-D scale**

| **Variable name** | **Variable label** | **Question text** | **Response options** |
| --- | --- | --- | --- |
| euro1 | Depression | In the last month, have you been sad or depressed? | 1=Yes; 0=No |
| euro2 | Pessimism | What are your hopes for the future? | 1=Any hopes mentioned; 0=No hopes mentioned |
| euro3 | Suicidality | In the last month, have you felt that you would rather be dead? | 1=Any mention of suicidal feelings or wishing to be dead; 0=No such feelings mentioned |
| euro4 | Guilt | Do you tend to blame yourself or feel guilty about anything? | 1=Obvious excessive guilt or self-blame; 0=No such feelings |
| euro5 | Sleep | Have you had trouble sleeping recently? | 1=Trouble with sleep pr recent change in pattern; 0=No trouble sleeping |
| euro6 | Interest | In the last month, what is your interest in things? | 1=Less interest than usual mentioned; 0=No mention of loss of interest |
| euro7 | Irritability | Have you been irritable recently? | 1=Yes; 0=No |
| euro8 | Appetite | What has your appetite been like? | 1=Diminution in desire for food; 0=No diminution in desire for food |
| euro9 | Fatigue | In the last month, have you had too little energy to do the things you wanted to do? | 1=Yes; 0=No |
| euro10 | Concentration | How is your concentration? For example, can you concentrate on a television program, film or radio program? Can you concentrate on something you read? | 1=Difficulty in concentrating on entertainment; 0=No such difficulty mentioned |
| euro11 | Enjoyment | What have you enjoyed doing recently? | 1=Fails to mention any enjoyable activity; 0=Mentions ANY enjoyment from activity |
| euro12 | Tearfulness | In the last month, have you cried at all? | 1=Yes; 0=No |

**5. Data availability**

The data (OR) are available in the online repository: <https://github.com/ukolovae/RA_and_depressive_symptoms>
